# Evaluation of technical efficiency of some rain-fed cereal and legume crops production in Syria: does crisis matter?

**DOI:** 10.1186/s40066-022-00389-y

**Published:** 2022-10-01

**Authors:** Naji AlFraj, Alaa Hamo

**Affiliations:** 1 Department of Agricultural Economics and Extension, AlFurat University, AlHassakeh, Syria; 2 Department of Agricultural Economics, Damascus University, Damascus, Syria

**Keywords:** Technical efficiency, Data envelopment analysis (DEA), Rain-fed crops, Crisis, Syria

## Abstract

**Background:**

Syria is a developing country whose economy is still dominated by the agricultural sector. The agricultural sector is considered as the main source of food in Syria and a major source of employment and income generation. Food and agricultural policies in Syria focus heavily on achieving food security and improving its four pillars (availability, accessibility, stability and utilization). As a result, until 2011, a good progress has been attained in food availability. The food security situation deteriorated in Syria after 2011 crisis, with the number of people facing acute food insecurity rising from 7.9 million in 2020 to a staggering 12.4 million in 2021. This is the result of many shocks that the agricultural sector has been exposed to, such as the relative decrease in cultivated areas, high costs of production, reduced input availability including labour, prevailing violence, related damage to farm equipment, and abandoned land. In view of the changes that the agricultural sector has been exposed to in Syria as a result of the crisis, the study concerns measuring the technical efficiency of production of some rain-fed cereal and legume crops in Syria and comparing it in the pre and post-crisis period, which has started in 2011. A non-parametric (DEA) method is applied for measuring technical efficiency during the time period 2003–2010 (pre-crisis) and the period 2011–2018 (post-crisis) with censored regression (the tobit model) to investigate the determinants of technical efficiency. A *t* test is used to test the null hypothesis (H_0_) that there was no difference in technical efficiency of the production of studied crops before and after the crisis in Syria and the alternative hypothesis (H_a_) that there was a significant difference in technical efficiency.

**Results:**

The findings show low level of technical efficiency in the post-crisis period. The results verified differences in the technical efficiency of pre- and post-crisis period. The use of censored regression with dummy for crisis has shown negative and significant effect on technical efficiency of each of the durum wheat and lentil crops, while it had no significant effect on the other studied crops.

**Conclusions:**

This study can provide important information to the government to pursue a new policy for recovery and improving the agricultural production and productivity. There is an urgent need to adopt new policies that focus on providing production requirements in the form of low-interest loans, sustainable use of resources, providing support for the marketing process, and focusing on the export markets of some study crops (chickpeas and lentils). Government should improve agricultural extension services for farmers and encouraging them to adopt new technologies.

## Introduction

Agriculture plays an important role in economic development, particularly in the developing economies of all regions [1]. Agricultural labor productivity and land productivity in Asia has grown faster than in other developing regions [2]. Syria is located in Western Asia and characterized by a middle-income developing country with a diversified economy. The agricultural sector is one of the largest contributors to Gross Domestic Product (GDP) and it plays a major role in Syria’s economic development for achieving national food security, promoting Syrian trade and providing jobs for the rural people [3]. Agriculture accounts for 20% of GDP in 2011, with an annual growth rate of about 4% from 2010 to 2011 [4]. The contribution of the agricultural sector to the GDP decreased with the beginning of the crisis in Syria, reaching about 18% in 2012 [5]. However, the contribution of the agricultural sector decreased to about 15% of the GDP during 2016–2019 [6]. The agricultural sector in Syria plays a major role in the national economy because of its multiple contributions to the economic and social development process of the country comprising gross output (16–18%), production inputs (13–18%), employment (27% of total population), investment (8.6%), total trade (11% of total trade), the activities of marketing, processing and providing the raw materials necessary for agro-industries, achievement of food security and environment sustainability [7]. In addition to its main role in generating income for a principal part of the rural population, which constitutes about 41% of the Syrian inhabitants in the period 2017–2019 [6].

The inability to achieve efficiencies in crop production is one of the main factors hindering the exploitation of the full potential of innovative technologies, particularly in developing countries [8]. Although researchers in developing and developed countries have been interested in studying the technical efficiency of the agricultural sector since the late 1980s [9, 10]; there is significantly less work done on developing and transition countries [11]. Those interested in development economics and agricultural economics have studied the sources of productivity growth in various countries; they are dependent on new experimental techniques and motivated by the desire to assess the impact of agricultural policies implemented on improving agricultural productivity in developing countries [10].

Syria is characterized by dry and semi-arid climate. Some 40–70% of the winter crops depending on rainfall availability that is characterized by considerable fluctuations from year to year [12]. Food and agricultural policies in Syria focus heavily on both achieving food security and improving its four pillars (availability, accessibility, stability and utilization). As a result, until 2011, a good progress has been attained in food availability comprising total availabilities and per capita availabilities, access to food, income improvement and its distribution, stability of supplies and access, food trade, food utilization, food quality and food safety [13]. In general, there was a decline in the abundance of food in Syria during the period 2010–2015, as the percentage of self-sufficiency for the group of food grains decreased and amounted to about 74.8%. It is also noted that the availability of food per capita for the different food groups decreased during the period 2010–2013 [14]. The food security situation deteriorated in Syria, with the number of people facing acute food insecurity rising from 7.9 million in 2020 to a staggering 12.4 million (60 percent of the population) in 2021 [15, 16]. This is the result of multiple shocks, including the protracted conflict and insecurity, mass displacement, the impact of the COVID-19 pandemic, fuel shortages, devastating wildfires and other climate-induced shocks [15].

For the agricultural sector in Syria, especially during the crisis, there is hardly any published work on measuring the technical efficiency of the agricultural sector. Therefore, this study aimed to assess the impact of the Syria crisis on achieving technical efficiency in the production of some rain-fed cereal and legume crops, and to identify the main factors affecting them during the period 2003–2018.

The paper contained the following sections: the plant production in Syria is presented in section "Plant production in Syria". Section "The impact of Syria crisis on the agricultural sector" presents the impact of the crisis in Syria on the agricultural sector. Section Materials and methods shows the materials and methods. Section "Results and discussion" is dedicated to the results and discussion and section "Conclusion and Recommendations" shows the conclusion and recommendations.

## Plant production in Syria

Syria is located in the region, where agriculture was first practiced, around 8000–10,000 years ago in the fertile crescent between the Tigris and Euphrates rivers, where the most important agricultural crops such as wheat, barley, lentil, vetch were planted in this region [17, 18].

Syria is located between latitudes 32 and 37 north, and longitudes 36 and 42 east, in the lower part of the Asian continent. It extends between the coastal Mediterranean regions in the west, and the desert region bordering Iraq and Jordan in the south and southeast, and includes very diverse regions in terms of temperature, rainfall, soil properties and water resources [19].

Plant production in Syria can be divided into three groups: field crops, vegetables and fruits. The cultivation of field crops is the most important in terms of cultivated area and production. They include cereals, legumes, grazing crops and industrial crops [20]. Plant production policies in Syria focused on providing food at the national level (wheat, legumes, etc.) and achieving food security by increasing yields, production of products that have comparative and competitive advantages in line with the demand of both national and international markets, adoption of alternative crops programs and conservation of environment and natural resources [21].

The area evolution reflects the impact of horizontal expansion policies on the performance of plant production [22]. Table 4 (Appendix) shows the development of the rain-fed cropped area by plant groups that include crops, vegetables and fruit trees during the period 2003–2018. In general, the cropped area considerably decreased from 3244.53 thousand ha in 2003 to 2945.52 thousand ha in 2018. It is noted that the areas planted with crops and fruit trees are subject to small fluctuations, with a coefficient of variation of about (10.27, 7.90%) for each of them, respectively, when compared to the areas planted with vegetables. These differences may be attributed to the fact that cereal and legume crops (wheat, barley, chickpeas, lentils) are strategic crops that farmers are committed to planting according to the agricultural plan issued by MAAR. The government has developed encouraging policies represented in providing loans to farmers of these crops, and the government annually sets the official price for these crops, as these crops are sold to public sector institutions and companies. All of the aforementioned factors contribute effectively to protecting farmers from market fluctuations and motivate them to produce these crops, and thus the relative stability of the cultivated areas. While the cultivation of vegetables is affected by supply and demand in the markets. The farmer's decision is greatly affected by the price in the previous season, and this largely explains the fluctuation of the areas planted with vegetables in Syria.

**Fig. 1:**
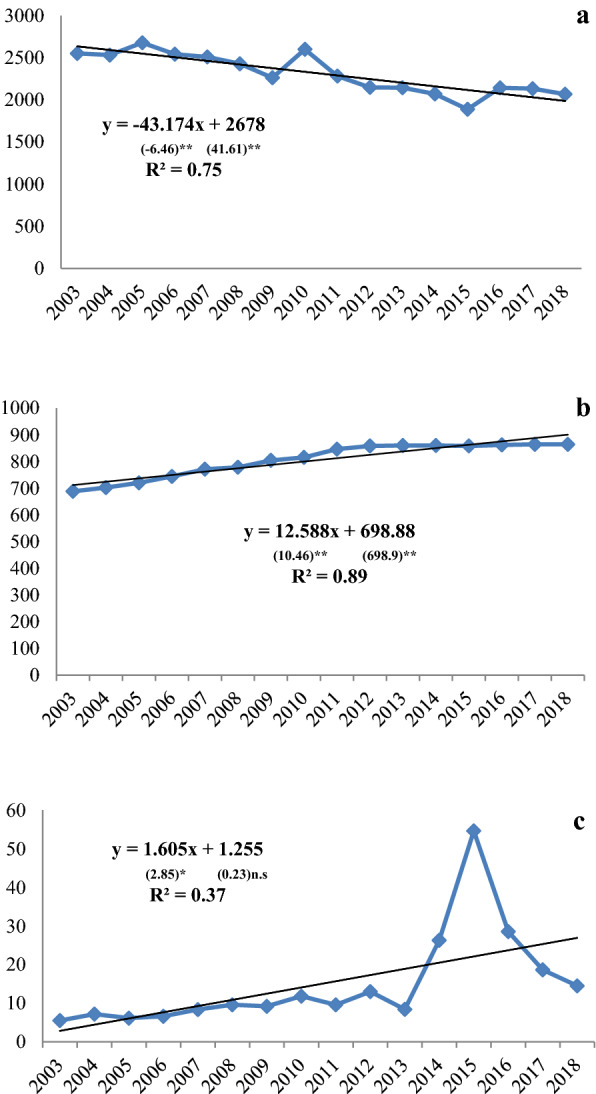
Linear trend of rain-fed cropped area (‘000 ha) by plant groups (**a** crops, **b** vegetables, and **c** fruits) in Syria, 2003–2018

The trend of the area planted with crops (Fig. 1a) in 2003–2018 shows a negative (decreasing) slope. The deficit trend of crops in Syria can be written in the equation as follows:

$$ Y = {2678 - 43}.{174}x. $$

The model has an estimate that every year, the area has decreased significantly by 43.174 thousand ha. According to FAO reports [23, 24], the areas planted with wheat in Syria declined in 2013 to about 1.418 million hectares, representing a 15 percent reduction compared with the average of 1.671 million hectares for the 10-year period 2002–2011. The area planted to barley is estimated to be 1.257 million hectares, this represents a reduction of about 6 percent from the 10-year average of 1.332 million hectares. Other reports indicated that estimated harvested wheat area in 2015 was the smallest since the 1960s. The total area under the Syrian Arab Republic’s principal legume crops appears to have changed very little since 2010.

Based on (Fig. 1b), it can be seen that visually, the trend of area planted with fruits tends to increase from year to year in the period 2003–2018. The trend of area planted with fruits can be written in the following equation:

$$ Y \, = {698}.{88} + {12}.{588}x. $$

From the equation above it can be seen that the regression coefficient has a positive sign, thus it gives an illustration that there is a positive relationship between the area variable and the time (year) variable. The regression coefficient value is 12.588, which means that there is an increase in the variable time (year) of one unit, which can cause an increase in planted area of 12.588 thousand ha. This may be due to the fact that most fruit trees (citrus in particular) has been produced in secure Government-held areas [23, 24].

The trend of area planted with vegetables in 2003–2018 shows also a positive (increasing) slop. In the linear trend graph, the vegetables area has the same model, *Y* = 1.255 + 1.605*x*. The model has an estimate that every year, the area has increased by 1.605 thousand ha.

**Fig. 2:**
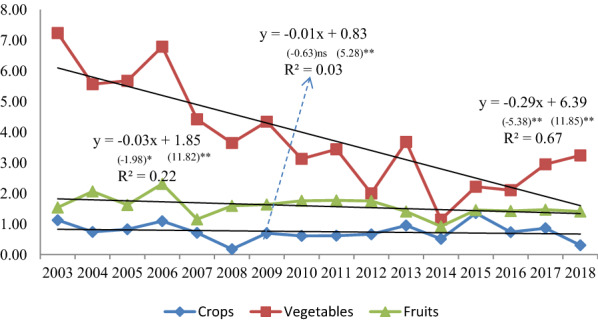
Linear trend of rain-fed yields (tons/ha) by plant groups in Syria, 2003–2018

The evolution of the yield reflects the impact of vertical expansion policies on the performance of plant production [22]. Table 4 (Appendix) gives an idea of the evolution of plant production yield according to the main groups during the period 2003–2018. It is noted that the variation in productivity during the period 2003–2018 was large, which negatively affects the availability of plant products. For more clarification, Fig. 2 shows the trend of productivity evolution, which indicates that it took a decreasing trend for crops, vegetables and fruit trees.

**Table 1:** Technical efficiency of some rain-fed cereal and legume crops production in Syria between 2003 and 2018

Crisis	Year	Soft wheat				Durum wheat				Barley				Lentils				Chick peas			
		TE^CRS^	TE^VRS^	SE	RTS	TE^CRS^	TE^VRS^	SE	RTS	TE^CRS^	TE^VRS^	SE	RTS	TE^CRS^	TE^VRS^	SE	RTS	TE^CRS^	TE^VRS^	SE	RTS
Pre-crisis	2003	1.000	1.000	1.000	–	0.925	0.957	0.967	irs	0.976	0.999	0.977	irs	0.875	0.963	0.909	irs	1.000	1.000	1.000	–
	2004	0.939	0.957	0.981	irs	0.985	1.000	0.985	irs	0.844	0.930	0.908	irs	1.000	1.000	1.000	–	0.940	0.943	0.997	irs
	2005	0.967	1.000	0.967	irs	0.957	0.977	0.979	irs	0.979	1.000	0.979	irs	1.000	1.000	1.000	–	1.000	1.000	1.000	–
	2006	0.788	1.000	0.788	irs	1.000	1.000	1.000	–	0.845	1.000	0.845	irs	0.966	1.000	0.966	irs	0.960	1.000	0.960	irs
	2007	0.582	0.977	0.596	irs	0.967	0.970	0.997	irs	0.799	0.913	0.875	irs	0.724	0.998	0.725	irs	0.884	1.000	0.884	irs
	2008	0.583	0.979	0.596	irs	0.906	0.912	0.994	irs	0.803	0.918	0.875	irs	0.725	1.000	0.725	irs	0.884	1.000	0.884	irs
	2009	0.596	0.865	0.689	irs	0.757	0.865	0.875	irs	0.599	0.881	0.679	irs	0.466	0.479	0.972	drs	0.520	0.632	0.823	irs
	2010	0.424	0.548	0.774	irs	0.562	0.589	0.953	irs	0.622	0.737	0.845	irs	0.566	1.000	0.566	drs	0.590	0.619	0.954	irs
	Mean	0.734	0.915	0.799	–	0.882	0.909	0.969	–	0.808	0.922	0.873	–	0.790	0.930	0.858	–	0.847	0.899	0.938	–
	SD	0.217	0.155	0.168	–	0.150	0.137	0.041	–	0.141	0.088	0.094	–	0.204	0.183	0.164	–	0.187	0.170	0.067	–
Post-crisis	2011	0.405	0.569	0.712	irs	0.570	0.597	0.954	irs	0.595	0.736	0.809	irs	0.589	0.610	0.965	drs	0.540	0.649	0.832	irs
	2012	0.435	0.549	0.792	irs	0.544	0.578	0.942	irs	0.493	0.639	0.772	irs	0.579	0.667	0.868	drs	0.528	0.572	0.923	irs
	2013	0.263	0.382	0.688	irs	0.348	0.378	0.920	irs	0.453	1.000	0.453	drs	0.512	0.528	0.970	drs	0.307	0.344	0.891	irs
	2014	0.212	0.296	0.716	irs	0.263	0.297	0.885	irs	0.346	0.476	0.727	drs	0.270	0.271	0.998	drs	0.249	0.288	0.866	irs
	2015	0.184	0.213	0.864	irs	0.227	0.240	0.948	irs	0.313	0.402	0.778	drs	0.255	0.276	0.925	drs	0.211	0.222	0.949	irs
	2016	0.101	0.156	0.647	irs	0.160	0.190	0.843	irs	0.229	0.509	0.451	drs	0.197	0.206	0.956	irs	0.161	0.187	0.862	irs
	2017	0.075	0.102	0.735	irs	0.102	0.131	0.777	irs	0.116	0.177	0.657	drs	0.099	0.101	0.989	irs	0.091	0.101	0.905	irs
	2018	0.056	0.075	0.747	irs	0.075	0.096	0.777	irs	0.089	0.130	0.688	drs	0.071	0.075	0.942	irs	0.058	0.065	0.891	irs
	Mean	0.216	0.293	0.738	–	0.286	0.313	0.881	–	0.329	0.509	0.667	–	0.322	0.342	0.952	–	0.268	0.304	0.890	–
	SD	0.144	0.192	0.066	–	0.189	0.191	0.074	–	0.180	0.287	0.141	–	0.210	0.230	0.041	–	0.183	0.211	0.037	–

TE^CRS^: Constant returns to scale technical efficiency, TE^VRS^: Variable returns to scale technical efficiency, SE: Scale Efficiency, irs: increasing returns to scale, drs: decreasing returns to scale, RTS: Type of returns to scale

Source: Own elaboration

## The impact of Syria crisis on the agricultural sector

Before the crisis, Syria’s economy was stable. The country embarked on a gradual economic liberalization to spur growth. Inflation was low and growth robust [25].

Prior to the beginning of the current crisis in 2011, agriculture played a very important part in Syria’s economy [26]. At the beginning of the 1990s, the agricultural sector contributed around 20% to (GDP), generated between 15% (in a dry year) and 40% (in a good year) of non-oil exports, and occupied at least 25% of the labor force [27]. The current crisis has devastated the agricultural sector. FAO estimates indicated that agriculture contributed to less than 17 percent of GDP in 2011, down from 20.4 percent in 2007 [24]. According to the estimates of the Central Bureau of Statistics (CBS), the number of workers in the agricultural sector decreased by about 535,999 workers, and they constituted about 12% of the total workforce of about 4,528,052 workers [5].

Syria is now in its eleventh year of crisis. Since 2011, the country’s economy has suffered hugely from the crisis. The crisis that Syria has experienced has damaged the national agricultural economy and the performance of Syrian agricultural trade, especially agricultural exports, and caused a drop in its returns [28]. The imports have been damaged as well due to their high cost comparing to the national currency which has been intensively dropped.

Agriculture-based livelihoods face severe constraints across the value chain—from production to market. Major constraints include: restricted access to land due to violence, internal population displacement, reduced availability and increased cost of farming inputs (seeds, fertilizers, animal feed, veterinary supplies, etc.), damage to farming equipment and infrastructure (including irrigation, storage and seed processing facilities) and limited veterinary supplies and services [25, 29].

Cereal production has been disrupted. Wheat production was estimated at 2.5 million tons in 2015, 20 percent lower than 2010. By contrast, barley, the next most important crop, has increased by 40 percent to 0.9 million tons since 2010 because of good rainfall [25]. FAO indicated in its special report [30] on its crop assessment in which it estimated Syria’s wheat production in 2021 as 1.05 million tons, compared to 2.8 million tons in 2020 and an average of 4.1 million tons (2002–2011). The 2021 barley crop was compromised by the lack of rainfall and only slightly over 20 percent of the rain fed area was actually harvested. Production is estimated at 268,000 tons, just about 12 percent of the 2020 output. Small barley harvests (below 500,000 tons) have not been unusual in the country and occurred in years with unfavorable rainfall even before the crisis (e.g., 212,000 tons in 2000, 271,000 tons in 1989).

Fruit production has suffered from the felling of trees for firewood, and from shortages of pesticides and fertilizers. The marketing of agricultural products was also greatly affected, due to the difficulties that farmers experienced in marketing their products, due to the government’s loss of almost complete control over the roads and main production areas, which negatively affected the food supply [25]. After several years of damage, destruction, neglect and natural deterioration of trees, fruit production started to recover in 2019. Overall, in 2019, about 1.04 million hectares were under fruit tree cultivation, including 45,000 hectares under productive citrus trees and 690,000 hectares under productive olive trees [30].

The last comprehensive livestock census, conducted in 2010, put the main livestock species at 18 million sheep, 2.3 million goats, 1.1 million cattle, 7000 buffaloes and 26.2 million poultry. In the first years of the crisis, it was evident that the livestock population had shrunk considerably. Sheep numbers fell by 45 percent, goat numbers by 30 percent, cattle numbers by 40 percent and poultry numbers by 55 percent. After a significant decline during the first years of the crisis, overall numbers of animals appeared to increase slightly or stabilize during 2016 and 2017, with 2017 being considered as the threshold year. Despite the economic challenges, including the high cost of feed, the latest (as of 2020) livestock situation in the country is characterized by gradual stabilization [30].

From the above, we can note the significant and complex negative impact of the crisis in Syria on the agricultural sector, which is one of the pillars of the Syrian economy, and the consequent challenges facing the country, such as restoring local food production and the rest of agricultural products, restoring marketing systems, and rebuilding the necessary infrastructure for production.

## Materials and methods

The data used in this study was obtained from the annual agricultural statistical collection issued by the Ministry of Agriculture and Agrarian Reform (MAAR) in Syria, and is available on the official website of the Ministry.

The technical efficiency of the production of some rain-fed cereal and legume crops (soft wheat, durum wheat, barley, lentils and chick peas) in Syria was calculated during the time period 2003–2010 (pre-crisis) and the period 2011–2018 (post-crisis) using Data Envelopment Analysis (DEA), via the software DEAP 2.1.

The DEA method is a non-parametric method, proposed by [31] with the aim of evaluating the relative efficiencies of comparable production units (DMUs) by means of variety of mathematical programming models.

Productivity and economic efficiency analysis has witnessed a new stage, since data envelopment analysis (DEA) was used as a non-parametric method in measuring technical and allocative efficiency [32]. Data Envelopment Analysis (DEA) is a very powerful service management and benchmarking technique originally developed by Charnes et al. [31] to evaluate nonprofit and public sector organizations [33]. This method is widely used by researches to analyze the performance of agricultural sector starting from different inputs and outputs [34].

The concept of technical efficiency was first proposed by Debreu (1951, 1959), then in 1962 Farrell and Fieldhouse formulated a linear programming model to measure the level of so‐called technical efficiency [35]. Technical efficiency means the ability of a DMUs (decision-making units) to obtain the largest possible amount of production using the available amounts of inputs [36, 37].

Technical efficiency is measured either assuming a constant return to scale (CRS); that is, all production units are operating at an optimum level, or assuming a variable return to scale (VRS); that is, the units of production are not operating at an optimum level. Technical efficiency can be measured with DEA by two approaches: (i) input-oriented model, which measures how many inputs could be reduced while maintaining the existing level of output, or (ii) output-oriented model, which measures how much output could be increased while using the given amount of inputs.

The output-oriented BCC model proposed by [38] was employed. This model assumes variable returns to scale (VRS) [39, 40]:

$$\mathrm{max}\varnothing $$

s.t.:

$$\sum_{j=1}^{n}{y}_{rj}{\lambda }_{j}\ge {\varnothing y}_{r0}$$

$$\sum_{j=1}^{n}{x}_{ij}{\lambda }_{j}\le {x}_{i0}$$

$$\sum_{j=1}^{n}{\lambda }_{j}=1$$

$$\lambda \ge 0$$

where DMU_o_ represents one of the *n* DMUs under evaluation, 1 ≤  Ø < ∞ and Ø − 1 is the proportional increase in output that could be achieved by the *i*th DMU_o_ with input quantities held constant, 1/Ø defines an output-oriented TE which varies between zero and one, *x*_*io*_ and *y*_*ro*_ are the *i*th input and *r*th output for DMU_o_, respectively; *i* is the number of inputs (*i* = 1, 2, …,*m*); *r* is the number of outputs (*r* = 1, 2, …, *n*); λ is the DMU’s weight and the efficiency score is Ø.

A measure of SE can be obtained by comparing the TE^CRS^ and TE^VRS^ scores. Any difference between the two TE scores indicates that there is scale inefficiency that limits the achievement of an optimal (constant) scale, which can be calculated as follows [39]:

$$ {\text{SE}}_{i} = {\text{TE}}_{i}^{{{\text{CRS}}}} /{\text{TE}}_{i}^{{{\text{VRS}}}} 0 \,\quad \le {\text{ SE}}_{i} \le { 1} $$

where SE_*i*_ = 1 indicates full-scale efficiency and SE_*i*_ < 1 indicates the presence of scale inefficiency.

The study tested the null hypothesis (H_0_) that there was no difference in technical efficiency of the production of some rain-fed cereal crops before and after the crisis in Syria and the alternative hypothesis (H_a_) that there was a significant difference in technical efficiency. A *t* test statistic was applied to verify if the TE scores of the two periods were significantly different. If a statistically significant difference was found, it would mean that there is no technological homogeneity between the two periods [41].

To investigate the determinants of technical efficiency, the study used censored regression model (the tobit model). Since the TE scores obtained from the VRS DEA model have values at the interval [0,1] the OLS estimates may be biased as well as inconsistent [42]. Similarly, Tobit regression was used by other researchers as well: Ahmed et al. [43] in mixed crop-livestock farming systems in Egypt, Sarker and Alam [44] in cotton production of Bangladesh, Dalgic et al. [45] in sheep farming of Turkey, Todorovic’ et al. [11] in arable farms of Serbia, and Nowak et al. [40] in European Union agriculture.

The general formulation is usually given in terms of an index function [46]:

$$ y_{i}^{*} = {\text{ x}}_{i} \prime \beta + \varepsilon_{i} , $$

$$ y_{i} = \, 0 \, \;{\text{if}}\;y_{i}^{*} \le \, 0, $$

$$ y_{i} = y_{i}^{*} \;{\text{if}}\;y_{i}^{*} > \, 0. $$

where *i* = 1,…, *N* indicates the observation; *y*_*i*_ are the realized or actual values of technical efficiencies; *y*_i_^*^ is a latent variable representing the TE scores obtained from the VRS DEA model; *x*_i_′ is a vector of explanatory variables described below; *β* is the coefficient parameter; and ε_*i* ~_*N(0,,σ*^*2*^*)* is an disturbance term*.*

To examine the factors affecting TE scores, six independent variables are introduced and analyzed in this study including number of tractors, fertilizer quantity, loans, number of employees, cultivated area, and crisis (post-crisis/pre-crisis). The specification of the empirical model is given by

$$ {\text{TE}}_{i}^{{{\text{VRS}}}} = \beta_{0} + \beta_{{1}} {\text{Ln }}\left( {{\text{tractors}}} \right) + \beta_{{2}} {\text{Ln }}\left( {{\text{fertilizer}}} \right) + \beta_{{3}} {\text{Ln }}\left( {{\text{loans}}} \right) + \beta_{{4}} {\text{Ln }}\left( {{\text{employees}}} \right) + \beta_{{5}} {\text{Ln }}\left( {{\text{area}}} \right) + \beta_{{6}} {\text{Crisis}} + \varepsilon_{i} $$

where tractors: number of tractors; fertiliser: the sum of nitrogen, phosphorous and potash content of various fertilisers consumed (tons); loans: value of agricultural loans (1000 Syrian pounds); employees: number of employees working in the Ministry of Agriculture and Agrarian Reform; Area: area cultivated in (hectares); Crisis: dummy variable (1 = post-crisis, 0 = pre-crisis).

It is worth noting that the data on the numbers of tractors, fertilizers, loans and employees were used for entire country. This is due to the fact that it is not available for each crop separately.

## Results and discussion

Efficiencies were calculated by the output-oriented data envelopment analysis model with the assumption of constant (CRS) and variable return to scales (VRS).

The results of efficiency scores, means and standard deviations of whole calculated scores are summarized in Table 1. The most important thing that can be observed in the results presented in Table 1 is that the technical efficiency of all studied crops witnessed a clear decline between 2003 and 2018. The difference between the most and the least efficient year was 92.5%, 90.4%, 87%, 92.5%, 93.5% for soft wheat, durum wheat, barley, lentils and chickpeas, respectively. The results show that the production of all the studied crops was characterized by full technical efficiency in 2006, because the ratio of the total technical efficiency was equal to 1. The minimum estimate is 0.075, 0.096, 0.130, 0.075, 0.065 for soft wheat, durum wheat, barley, lentils and chickpeas, respectively in 2018. This means that improving the capabilities of the agricultural sector in Syria, which has deteriorated after the crisis, can increase the production of these crops by 87 to 93.5% without any increase in the amount of economic resources used.

The findings show that the scale efficiency (SE) was found, on average, to be equal to 0.799, 0.969, 0.873, 0.858, 0.938 implying that production could increase by about 20%, 3%, 13%, 14%, 6% for soft wheat, durum wheat, barley, lentils, and chick peas, respectively, in the pre-crisis period. As for the scale efficiency in the post-crisis period, it was found, on average, to be equal to 0.738, 0.881, 0.667, 0.952, 0.890 implying that production could increase by 5–33%.

Table 1 reports the returns to scale (RTS), indicating that all studied crops except barley and lentils exhibiting an increasing returns to scale in the pre- and post-crisis period, implying that the production of these crops needs to be expanded to achieve full scale efficiency. By contrast, barley and lentil crops in the post-crisis period in particular exhibiting a decreasing returns to scale. These findings suggest that the production of these crops should be reduced to reach the optimal scale. However, we can note from the results of the table that all crops in the pre-crisis period, except for barley, revealed in some years an optimal level of scale efficiency.

Table 2 exhibits the results of test of difference of means, where the *t* test was performed. The *p* values of the *t* tests indicate that the differences between TE^VRS^ and TE^CRS^ means are significant between the pre- and post-crisis period. This indicates the negative impact caused by the crisis in Syria since 2011. In other words, the production of studied crops operate on different technological grounds (no technological homogeneity) [41].

**Table 2:** Test of difference of means

Indicators	Crop production	Hypothesis	Difference	*t* test		Decision
				*t*-statistic	*p* value	
TE^**VRS**^	Soft Wheat	H_0_: diff = 0 H_a_: diff ≠ 0	0.623	10.649	.000	Reject H_0_
	Durum Wheat		0.595	11.107	.000	Reject H_0_
	Barley		0.413	5.216	.001	Reject H_0_
	Lentils		0.588	7.221	.000	Reject H_0_
	Chick peas		0.595	9.639	.000	Reject H_0_
TE^CRS^	Soft Wheat	H_0_: diff = 0 H_a_: diff ≠ 0	0.518	13.526	.000	Reject H_0_
	Durum Wheat		0.596	11.129	.000	Reject H_0_
	Barley		0.479	17.794	.000	Reject H_0_
	Lentils		0.468	10.970	.000	Reject H_0_
	Chick peas		0.579	12.173	.000	Reject H_0_
SE	Soft wheat	H_0_: diff = 0 H_a_: diff ≠ 0	0.061	0.919	.389	Accept H_0_
	Durum wheat		0.088	4.385	.003	Reject H_0_
	Barley		0.206	3.347	.012	Reject H_0_
	Lentils		0.093	1.621	.149	Accept H_0_
	Chick peas		0.047	1.579	.158	Accept H_0_

Source: Own elaboration

Table 3 presents the results of an estimation of the Tobit model coefficients. The findings indicate that number of tractors positively and significantly affect the technical efficiency of production of lentils (*p* value < 0.10). On average, if number of tractors increases by one, this will increase the technical efficiency of production of lentils by 7.148%. This result was in line with the findings of Vortia et al. [47] and Tun and Kang [48] who revealed a significant positive effect of machines on technical efficiency. While it negatively and significantly affected the technical efficiency of soft wheat (*p* value < 0.10). This can be attributed to the fact that the majority of wheat farmers are mainly rely on hiring machinery during the entire production period. This result conforms to the findings of Baba et al. [49] who showed that uprooting machine negatively influences the cassava yield and producers’ technical efficiency in Cambodia. Njeru [50] also revealed a significant negative effect of hiring of capital equipment on the level of technical efficiency. The results were insignificant for durum Wheat, barley, and Chick peas. Fertilizer quantity had a positive and significant effect on the level of technical efficiency for most crops. For instance, the effect was significant at the 1% level for each of soft wheat, durum wheat and chick peas, and at the 5% level for lentils. The significant positive coefficient of fertilizer quantity indicates that the technical efficiency for production of these crops increases significantly with increase in fertilizer quantity. Similar findings have been reported by Chaudhry [51] who found that total fertilizer nutrients applied as well as the balanced mix of nutrients affect technical efficiency positively. Salam et al. [52] also found a similar relation. While the effect was not significant for barley.

**Table 3:** Determinants of technical efficiency of rain-fed crop production in Syria: Tobit regression

Variables	Crops				
	Soft wheat	Durum wheat	Barley	Lentils	Chick peas
Constant	31.015^**^ (2.34)	0.777^n.s^ (0.05)	19.530 ^n.s^ (0.59)	− 76.273^**^ (− 2.28)	15.528^n.s^ (0.63)
Ln number of tractors	− 2.811^*^ (− 2.01)	− 0.802^n.s^ (− 0.56)	− 3.935 ^n.s^ (− 1.04)	7.148^*^ (2.12)	− 1.467^n.s^ (− 0.58)
Ln fertilizer quantity	0.234^***^ (6.03)	0.202^***^ (7.36)	0.133 ^n.s^ (1.81)	0.153^**^ (2.41)	0.223^***^ (3.01)
Ln loans	− 0.017^*^ (− 2.11)	− 0.014^*^ (− 2.15)	− 0.035^*^ (− 1.96)	− 0.044^**^ (− 3.04)	− 0.017 ^n.s^ (− 1.33)
Ln number of employees	0.199^n.s^ (0.52)	0.298^n.s^ (0.89)	0.423^n.s^ (0.49)	− 1.350^n.s^ (− 1.73)	− 0.144^n.s^ (− 0.19)
Ln soft wheat area	− 0.183 ^n.s^ (− 1.07)	–	–	–	–
Ln durum wheat area	–	0.295^n.s^ (1.62)	–	–	–
Ln barley area	–	–	1.505^n.s^ (1.69)	–	–
Ln lentils area	–	–	–	0.701^**^ (2.28)	–
Ln chick peas area	–	–	–	–	0.133^n.s^ (0.52)
Crisis^a^	− 0.183^n.s^ (− 1.69)	− 0.219^**^ (2.31)	0.177^n.s^ (0.55)	− 0.797^***^ (− 3.29)	− 0.195^n.s^ (− 1.16)
Sigma	0.065	0.060	0.159	0.127	0.119
Log-likelihood	12.306	15.345	1.386	3.103	2.966

Source: Own elaboration

^***^Significant at 1.0% level, ^**^Significant at 5.0% level, ^*^Significant at 10.0% level, ^a^Dummy variable (1 = post-crisis, 0 = pre-crisis)

Figures in parentheses are the *t* value

Surprisingly, the result indicates that the loans was negatively and significantly associated with level of technical efficiency for soft wheat, durum wheat, barley (*p* value < 0.10), and lentils (*p* value < 0.05) implying that technical efficiency is lower on farms that receive the loans. This may be due to the misuse of these loans and allocating part of them for non-agricultural purposes. Chaudhry [51] and Salam et al. [52] also found similar results.

The number of employees had a positive effect on efficiency for soft wheat, durum wheat and barley. While the effect was negative for lentils and chick peas. However, the estimated coefficients were not significant. The cultivated area had no significant effect on the levels of technical efficiency, except for the lentil crop, which was positively and significantly affected by the cultivated area. The significant positive coefficient of area indicates that the technical efficiency for production of lentil increases significantly with increase in cultivated area. Similar findings have been reported by Majumder et al. [53] and Tan et al. [54], although there are some studies reporting contradictory results Houngue and Nonvide [55]. The findings indicate that the crisis negatively and significantly affected the levels of technical efficiency of each of the durum wheat and lentil crops, while it had no significant effect on the other studied crops.

## Conclusion and recommendations

This study dealt with an important topic regarding the agricultural sector in Syria, which has been witnessing difficult conditions since 2011. It is also one of the rare studies that dealt with this aspect. In this study, we tried to answer the following question: “Has the Syria crisis, which began in 2011, affected the levels of technical efficiency of some rain-fed cereal and legume crops production?”. This study has measured the technical efficiency of production of some rain-fed cereal and legume crops (soft wheat, durum wheat, barley, lentils and chick peas) using data for the period (2003–2018).

It has been found that the production of all the studied crops was characterized by full technical efficiency in 2006, while the minimum levels of technical efficiency were achieved in 2018. The results indicate that the level of the technical efficiency of crops production is diverse between pre and post-crisis period and witnessed a clear decline between 2003 and 2018. With regard to the factors determining technical efficiency, the crisis variable had a significant negative impact on the levels of technical efficiency for both durum wheat and lentil crops. Regarding the fertilizer quantity, we found that it had a positive statistically significant impact on the technical efficiency. Tractors had positive impact on technical efficiency of lentils, while it negatively and significantly affected the technical efficiency of soft wheat. In contrast, number of employees had an insignificant impact on the technical efficiency of all crops. Our results also suggest unexpectedly that the provision of agricultural loans had a negative impact on technical efficiency.

The results of this study can provide important information for the government to pursue a new policy to recovery and reconstruction of the agricultural sector, where it seems that there is an urgent need to give the agricultural sector great importance by increasing agricultural investments. These investments must also bring with them the ability to innovate and adopt new technologies, as most of the studied crops exhibiting an increasing returns to scale in the pre and post-crisis period, implying that the production of these crops need to be expanded to achieve full scale efficiency. Such as developing agricultural research and extension services that focus on maintaining and sustaining resources affected by the crisis, increasing productivity by providing improved seeds with the aim of increasing production and achieving food security, and providing appropriate technical training that enables farmers to use resources effectively through the rehabilitation of damaged extension units. In addition, the government should adopt the required legislation to encourage investment in the agricultural sector and benefit from donor countries and international organizations, such as issuing laws that would facilitate investment and simplify its procedures, and not impose large taxes and fees on agricultural investments.

## Data Availability

Available upon request.

## Appendix

See Table 4.

**Table 4:** Area, yield and production of plant groups (crops, vegetables, and fruits) in Syria, 2003–2018

Item	Area (‘000 ha)			Yield (t/ha)			Production (‘000 t)		
	Crops	Vegetables	Fruits	Crops	Vegetables	Fruits	Crops	Vegetables	Fruits
2003	2551	5.53	688	1.12	7.23	1.54	2869	40	1057
2004	2531	7.19	702	0.74	5.56	2.06	1870	40	1447
2005	2678	6.17	720	0.82	5.67	1.62	2207	35	1166
2006	2542	6.63	744	1.09	6.79	2.31	2772	45	1718
2007	2509	8.38	771	0.71	4.42	1.15	1779	37	888
2008	2428	9.61	778	0.18	3.64	1.59	427	35	1237
2009	2262	9.22	804	0.70	4.34	1.63	1575	40	1313
2010	2600	11.84	815	0.61	3.13	1.75	1582	37	1430
2011	2282	9.6	846	0.62	3.44	1.77	1405	33	1496
2012	2148	13.03	858	0.66	2.00	1.75	1423	26	1499
2013	2144	8.43	860	0.95	3.68	1.40	2035	31	1208
2014	2070	26.32	860	0.51	1.14	0.92	1061	30	792
2015	1888	54.64	858	1.35	2.21	1.45	2551	121	1246
2016	2143	28.57	862	0.73	2.10	1.42	1570	60	1225
2017	2133	18.66	864	0.86	2.95	1.46	1829	55	1265
2018	2067	14.52	864	0.30	3.24	1.41	628	47	1214
Mean	2311.00	14.90	805.88	0.75	3.85	1.58	1723.94	44.50	1262.56
Standard deviation	237.29	12.62	63.65	0.29	1.74	0.33	685.08	22.26	231.43
CV%^*^	10.27	84.71	7.90	39.47	45.20	20.72	39.74	50.01	18.33

Source: MAAR 2018 [56], ^*^ CV = standard deviation/mean × 100

## Abbreviations

DEA: Data Envelopment Analysis
DMU: Decision-making units
GDP: Gross domestic product
MAAR: Ministry of Agriculture and Agrarian Reform
FAO: Food and Agriculture Organization of the United Nations
CBS: Central Bureau of Statistics
CRS: Constant return to scale
VRS: Variable return to scale
TE: Technical efficiency
SE: Scale efficiency
OLS: Ordinary least-squares
RTS: Returns to scale
irs: Increasing returns to scale
drs: Decreasing returns to scale
